# Nalidixic acid potentiates the antitumor activity in sorafenib-resistant hepatocellular carcinoma *via* the tumor immune microenvironment analysis

**DOI:** 10.3389/fphar.2022.952482

**Published:** 2022-08-22

**Authors:** Zhi-Yong Liu, Dan-Ying Zhang, Xia-Hui Lin, Jia-Lei Sun, Weinire Abuduwaili, Guang-Cong Zhang, Ru-Chen Xu, Fu Wang, Xiang-Nan Yu, Xuan Shi, Bin Deng, Ling Dong, Shu-Qiang Weng, Ji-Min Zhu, Xi-Zhong Shen, Tao-Tao Liu

**Affiliations:** ^1^ Department of Gastroenterology and Hepatology, Zhongshan Hospital of Fudan University, Shanghai, China; ^2^ Shanghai Institute of Liver Disease, Shanghai, China; ^3^ Department of Gastroenterology, The Affiliated Hospital of Yangzhou University, Yangzhou, China; ^4^ Key Laboratory of Medical Molecular Virology, Shanghai Medical College of Fudan University, Shanghai, China

**Keywords:** immune gene signature, sorafenib resistance, tumor immune microenvironment, adjuvant drugs, bioinformatics analysis

## Abstract

Sorafenib resistance is often developed and impedes the benefits of clinical therapy in hepatocellular carcinoma (HCC) patients. However, the relationship between sorafenib resistance and tumor immune environment and adjuvant drugs for sorafenib-resistant HCC are not systemically identified. This study first analyzed the expression profiles of sorafenib-resistant HCC cells to explore immune cell infiltration levels and differentially expressed immune-related genes (DEIRGs). The prognostic value of DEIRGs was analyzed using Cox regression and Kaplan–Meier analysis based on The Cancer Genome Atlas. The primary immune cells infiltrated in sorafenib-resistant HCC mice were explored using flow cytometry (FCM). Finally, small-molecule drugs for sorafenib-resistant HCC treatment were screened and validated by experiments. The CIBERSORT algorithm and mice model showed that macrophages and neutrophils are highly infiltrated, while CD8^+^ T cells are downregulated in sorafenib-resistant HCC. Totally, 34 DEIRGs were obtained from sorafenib-resistant and control groups, which were highly enriched in immune-associated biological processes and pathways. NR6A1, CXCL5, C3, and TGFB1 were further identified as prognostic markers for HCC patients. Finally, nalidixic acid was identified as a promising antagonist for sorafenib-resistant HCC treatment. Collectively, our study reveals the tumor immune microenvironment changes and explores a promising adjuvant drug to overcome sorafenib resistance in HCC.

## Introduction

Hepatocellular carcinoma (HCC), the most common type of primary liver cancer, represents the sixth most common cancer and the fourth leading cause of cancer-related death worldwide (Bray et al., 2018; Siegel et al., 2021). Surgical resection and liver transplantation are still the mainstays in HCC treatment; however, the access depends on tumor size, tumor number, extrahepatic metastasis, and clinical status of patients (Roayaie et al., 2015; Kokudo et al., 2019). Sorafenib has been approved as first-line molecular-targeted therapy for advanced HCC patients (Llovet et al., 2008; Cheng et al., 2009; Kokudo et al., 2019), which attenuates cell proliferation by blocking the Raf-MEK-ERK pathway and arrests cell cycle by decreasing cyclin D1. Despite these impressive advances, overall survival remains dismal in sorafenib-treated HCC patients, with median overall survival increased from 4.2 to 6.5 months (Llovet et al., 2008; Keating, 2017). Moreover, many advanced HCC patients developed resistance to sorafenib and exhibited poor prognosis (Lin et al., 2020; Xia et al., 2020). Previous research suggested multiple mechanisms were involved in the development of sorafenib resistance, such as RNA N6-methyladenosine (Lin et al., 2020), cancer stem cell (Leung et al., 2021), hypoxia microenvironment (Wu et al., 2020), and ferroptosis (Sun et al., 2016). While the mechanism of sorafenib resistance in HCC patients remains poorly understood, investigating the underlying mechanisms is still of great significance in developing novel therapeutic strategies and exploring promising adjuvant drugs for HCC patients.

Tumor immune microenvironment (TIME), as a contributory part to regulating the progression of tumors, includes mainly tumor-infiltrating lymphocytes and other assorted immune cells, such as T cells, macrophages, natural killer cells, and dendritic cells (Lei et al., 2020). The immune system functions to constantly observe and eliminate pre-cancerous cells to prevent the progression to tumor. However, suppression of the immune system contributes to tumor escape and progression (Marzagalli et al., 2019). Recently researchers have also found that immunocytes play critical roles in tumor development and chemotherapy response by cross-talking with tumor cells, including tumor-associated neutrophils (Zhou et al., 2016), natural killer cells (Sprinzl et al., 2013), T cells (Zhou et al., 2016), regulatory T cells (Granito et al., 2021), and tumor-associated macrophages (Zhang et al., 2010; Zhou et al., 2016). Although the biological mechanisms of sorafenib resistance have been explored extensively, the relationship between sorafenib resistance and the TIME in HCC remains to be elusive.

Given limited investigation determining the sorafenib-resistant TIME during HCC development and progression, this study aimed to evaluate the relationship between sorafenib resistance and the TIME and explore a promising drug to overcome sorafenib resistance in HCC patients. Our results suggest that NR6A1, CXCL5, C3, and TGFB1 are critical DEIRGs in sorafenib-resistant cells, which are markedly associated with the survival time of HCC patients and infiltration levels of immune cells. NAL may serve as an adjuvant drug for sorafenib-resistant HCC treatment.

## Materials and methods

### Data acquisition

The transcriptional data of three parental HCC and five sorafenib-resistant HCC xenografts in the GEO dataset (GSE121153) were obtained for immune cell infiltration analysis. Gene expression data of sorafenib-resistant and control HCC cells were downloaded from the GEO dataset (GSE94550) for gene expression differential analysis. Immune-related genes (IRGs) were obtained from the Immunology Database and Analysis Portal (ImmPort; http://www.immport.org/) (Bhattacharya et al., 2018). Transcriptome sequencing profiles and clinical characteristics of LIHC (liver hepatocellular carcinoma) for differentially expressed gene (DEG) examination and survival analysis were obtained from the TCGA GDC data portal (https://portal.gdc.cancer.gov/), including 371 LIHC and 86 non-LIHC tissue samples.

### Immune cell infiltration analysis by CIBERSORT

CIBERSORT is an analytical tool to estimate the composition of member cell types in a mixed cell population by gene expression profiles (https://cibersort.stanford.edu/) (Newman et al., 2015). The “CIBERSORT R” package with LM22, a leukocyte gene signature matrix including 22 human immune cell types, was applied to analyze immune cell infiltration among parental and sorafenib-resistant HCC xenografts in the GSE121153 dataset.

### Gene set enrichment analysis in sorafenib-resistant hepatocellular carcinoma cells

Gene set enrichment analysis (GSEA) analysis was performed using GSEA software (V4.1.0) with Gene Ontology (GO) and Kyoto Encyclopedia of Genes and Genomes (KEGG) pathway gene sets between sorafenib-resistant and control HCC cells (GSE94550) to explore different biological functions (Subramanian et al., 2005).

### Differential expression analysis between sorafenib-resistant and control hepatocellular carcinoma cells

The “limma R” package was performed to analyze the microarray data between sorafenib-resistant and control HCC cells. The threshold of adjusted *p* < 0.05 and absolute fold change (log2) > 1 was established to screen DEGs. DEIRGs were obtained from overlapped DEGs based on the ImmPort database. The volcano plots and heat maps were presented by R.

### Functional enrichment analyses for differentially expressed immune-related genes

To explore the functions among DEIRGs, Gene Ontology (GO) functional annotations and KEGG pathway enrichment analysis were conducted using the Database for Annotation, Visualization, and Integrated Discovery (DAVID: https://david.ncifcrf.gov/) (Huang da et al., 2009). The immune-related GO terms of the GO Circle plot were performed by the “GOplot R” package (Walter et al., 2015). Furthermore, the KEGG pathway was plotted using the “ggplot2 R” package.

### Association and mutation analysis of differentially expressed immune-related genes

Pearson’s analysis of DEIRGs was carried out based on TCGA expression data using the “corrplot R” package. DEIRG mutation analysis in LIHC patients was performed using the “maftools R” package.

### Identification of prognosis-related differentially expressed immune-related genes

Univariate Cox regression analysis was conducted to discover potential prognostic biomarkers. The results were plotted by the “forestplot R” package. Those with *p* < 0.05 were selected as prognosis-related DEIRGs. The relationship between potential prognostic DEIRGs and critical targets of the sorafenib-related pathway was explored by Pearson’s analysis. Kaplan–Meier analysis was performed to verify the prognostic value of DEIRGs. In addition, the nomogram of prognosis-related DEIRGs, clinical characteristics, pathologic stage, and survival probability of TCGA-LIHC patients were plotted by the “rms” package of R software based on the Cox proportional hazard regression model.

### UALCAN analysis

UALCAN (http://ualcan.path.uab.edu) is a comprehensive online platform to explore cancer data and validate the genes of interest (Chandrashekar et al., 2017). UALCAN was used to analyze the relative expression of prognosis-related DEIRGs between normal samples and HCC patients of different clinicopathological stages in TCGA.

### Immune cell infiltration and prognosis-related differentially expressed immune-related gene expression

Tumor Immune Estimation Resource (TIMER) was used to analyze the association between tumor-infiltrating immune cells and prognosis-related DEIRGs. TIMER is a public database containing 32 types of cancers and 10,897 TCGA samples and provides a web tool for analysis and visualization of the six kinds of immunocyte infiltration in tumor samples (Li et al., 2017).

### Single-cell expression of prognosis-related differentially expressed immune-related genes in liver

The expression of prognosis-related DEIRGs in different liver cell types was explored in The Single-Cell-Type Atlas (https://www.proteinatlas.org/) (Uhlén et al., 2015). The Single-Cell-Type Atlas includes single-cell RNA sequencing (scRNA-seq) data of 13 human tissues. The scRNA-seq comprises 192 different cell type clusters in 12 main cell type groups. The expression of selected genes expressed in each cell type will be plotted using interactive Uniform Manifold Approximation and Projection for Dimension Reduction (UMAP).

### Identification of small bioactive molecules

DEIRGs between sorafenib-resistant and control groups were analyzed to identify potential small molecules using the Connectivity Map (CMap) database. The CMap, a web-based tool, can be applied to predict the biochemical interactions of small molecules with disease-related gene signature, thus helping researchers find novel uses for existing drugs and understanding the molecular mechanisms of diseases (Lamb et al., 2006).

### Cell culture and sorafenib-resistant cell line establishment

The mouse HCC cell line Hepa1-6 and human HCC cell line Huh7 were purchased from the cell bank of the Chinese Academy of Science (Shanghai, China). The cells were cultured in Dulbecco’s modified Eagle’s medium (DMEM) (KeyGEN, Nanjing, China) with 10% fetal bovine serum (FBS) (Sigma, Saint Louis, United States) and 100 U/ml of penicillin and 50 μg/ml strepomycin (Gibco, California, United States) in a 37 °C humidified incubator containing 5% CO_2_. Sorafenib-resistant cell lines were established as previously described (Jiang et al., 2018; Lu et al., 2018; Xu et al., 2020). Hepa1-6 and Huh7 cells were cultured in DMEM containing 1 μM sorafenib for 2 weeks. The sorafenib concentration of the culture medium is increased slowly by 0.5 μM per week for 4–5 months until the cells can survive in 10 μM sorafenib concentration. The sorafenib-resistant cell lines Hepa1-6 and Huh7 were obtained, termed Hepa1-6-SR and Huh7-SR, and continuously cultured in DMEM with sorafenib.

### RNA extraction, reverse transcription, and qRT-PCR

Total RNA was extracted by TRIzol reagent (Takara, Japan). Reverse transcription was performed using 1 μg RNA by the Hifair^®^ Ⅱ 1st Strand cDNA Synthesis Kit (Yeasen, Shanghai, China). The qRT-PCR assay was conducted by Hieff^®^ qPCR SYBR Green Master Mix (Yeasen, Shanghai, China) according to the manufacturer’s instruction. β-actin was used as an internal control, and the relative expression levels of target genes were analyzed using the 2^−ΔΔCT^ method. The PCR primers used in this study are listed in Supplementary Table S1.

### Cell viability assay

Cell viability was assessed using the cell counting kit 8 (CCK-8) (Beyotime, Shanghai, China) according to the manufacturer’s protocol. 1 × 10^4^ HCC cells per well were cultured in 96-well plates and treated with different concentrations of sorafenib with or without nalidixic acid (NAL). After incubation at 37°C for indicated times, 10 μL of CCK-8 medium in each well was added to the cells and incubated for additional 60 min. The absorbance was determined at 450 nm using a spectrophotometer (Thermo Fisher, California, United States).

### Half-maximal inhibitory concentration (IC_50_) assay

Sorafenib-resistant cells were cultured in 96-well plates in 1×10^4^ cells per well with fresh medium containing nalidixic acid (0, 1, 5, 10, 20, and 50 μg/ml). After 48 h incubation at 37°C, CCK-8 was used to evaluate cell viability by using a spectrophotometer (Thermo Fisher, California, United States) at 450 nm. The IC_50_ values were calculated by comparing the absorbance and inhibition rate.

### Tumor xenograft mouse model

All the mice were purchased from Shanghai Laboratory Animal Company (Shanghai, China) and fed in specific pathogen-free (SPF) conditions. The animal study was approved by the Animal Care and Use Committee at Zhongshan Hospital of Fudan University.

To establish a liver orthotopic xenograft mouse model, 5 × 10^6^ Hepa1-6 and Hepa1-6-SR cells were subcutaneously injected into the back flanks of two C57BL/6 mice. After 2 weeks, resecting the subcutaneous tumors and dissecting into 3-mm^3^ tissue masses in volume were carried out. Then, the tumor masses were planted into other mouse livers under anesthesia. The mice were euthanized after 4 weeks, and tumors were harvested and weighed.

To establish the subcutaneous xenograft mouse model, 5 × 10^6^ Hepa1-6 and Hepa1-6-SR cells were subcutaneously injected into the back flanks of C57BL/6 mice. After 2 weeks, the tumor-bearing mice were treated with sorafenib (30 μg/g/mouse; daily, oral gavage) and sorafenib combined nalidixic acid (50 μg/g/mouse; daily, oral gavage) for additional 2 weeks. The tumor length and width were recorded every 3 days. Tumor volumes were calculated as length × width^2^ × 0.5.

### Immunohistochemistry

Mouse tumor tissues were fixed in 4% paraformaldehyde and embedded in paraffin. After deparaffinization and rehydration, the tissue sections were subjected to immunohistochemistry (IHC) analysis. The images were captured using a light microscope (Olympus, Tokyo, Japan). The primary antibody Ki-67 (1:500, Abclonal, Wuhan, China) was used in this study.

### Multiplex immunofluorescence staining

Multiplex immunofluorescence (mIF) staining was performed using Opal 7-Color fIHC Kit (PerkinElmer, Waltham, United States) according to protocols which have been described previously (Parra et al., 2017; Parra et al., 2018). The embedded tumor tissues that underwent deparaffinization and rehydration were heated at 95°C for 20 min using Tris–EDTA buffer or citrate buffer to retrieve antigen. Next, the slides were incubated with primary antibodies overnight at 4°C. Then, the slides were washed three times with 2-methyl-2H-isothiazol-3-one and incubated with horseradish peroxidase (HRP)-conjugated secondary antibody for 10 min at room temperature. Next, the slides were incubated at room temperature for 10 min with one of the following Alexa Fluor tyramides included in the Opal 7 kit to detect antibody staining. After BOND Wash Solution washing, the slides were counterstained with DAPI for 5 min to visualize nuclei and then mounted with glycerine. The slides were scanned using the Pannoramic MIDI System (3DHISTECH, Budapest, Hungary). The following primary antibodies are used in this study: CD8 (1:50, Servicebio, Wuhan, China), Ly-6G (1:200, Servicebio, Wuhan, China), and F4/80 (1:200, Servicebio, Wuhan, China).

### Flow cytometry

Tumor tissues were resected from mice and minced into small pieces and then were lysed by 1 mg/ml collagenase IV (Sigma, United States) and DNase I (Invitrogen, United States) for 1 h at 37°C. Afterward, the tissue medium was filtrated using a 70-μm filter screen to obtain single-cell suspensions. The cell suspensions were stained with antibodies for 30 min and washed three times by PBS and then were subjected to FCM analysis. The following reagents and antibodies were used in FCM analysis.

Panel A: LIVE/DEAD™ Fixable Stain (Invitrogen, California, United States), anti-mouse CD45-BV605 (Biolegend, California, United States), anti-mouse CD3-PE-cy7 (Biolegend, California, United States), anti-mouse CD4-Efluor450 (BD, New Jersey, United States), anti-mouse CD8-Percp-cy5.5 (Biolegend, California, United States), anti-mouse CD19-BV650 (Biolegend, California, United States), and anti-mouse NK1.1-PE (Biolegend, California, United States).

Panel B: LIVE/DEAD™ Fixable Stain (Invitrogen, California, United States), anti-mouse CD45-BV605 (Biolegend, California, United States), anti-mouse CD11b-Percp-cy5.5 (Biolegend, California, United States), anti-mouse F4/80-PE (Biolegend, California, United States), and anti-mouse Ly6G-APC (Biolegend, California, United States).

### Statistical analysis

R software (v4.0.3), GraphPad Prism (v9.0), and SPSS (v21.0) were used for statistical analysis. All experiments were performed at least three times. Data were presented as mean ± SD. Unpaired Student’s t tests were used to analyze the difference between two groups. For different group comparison, one-way analysis of variance (ANOVA) was performed. *p* < 0.05 was considered statistically significant.

## Results

### The immune microenvironment analysis and differentially expressed immune-related gene identification in sorafenib-resistant hepatocellular carcinoma

While clinical practice using sorafenib monotherapy, or in combination with other agents, has been disappointing, it is worthwhile to understand why sorafenib is resistant to therapy using the tumor microenvironment.

**FIGURE 1:**
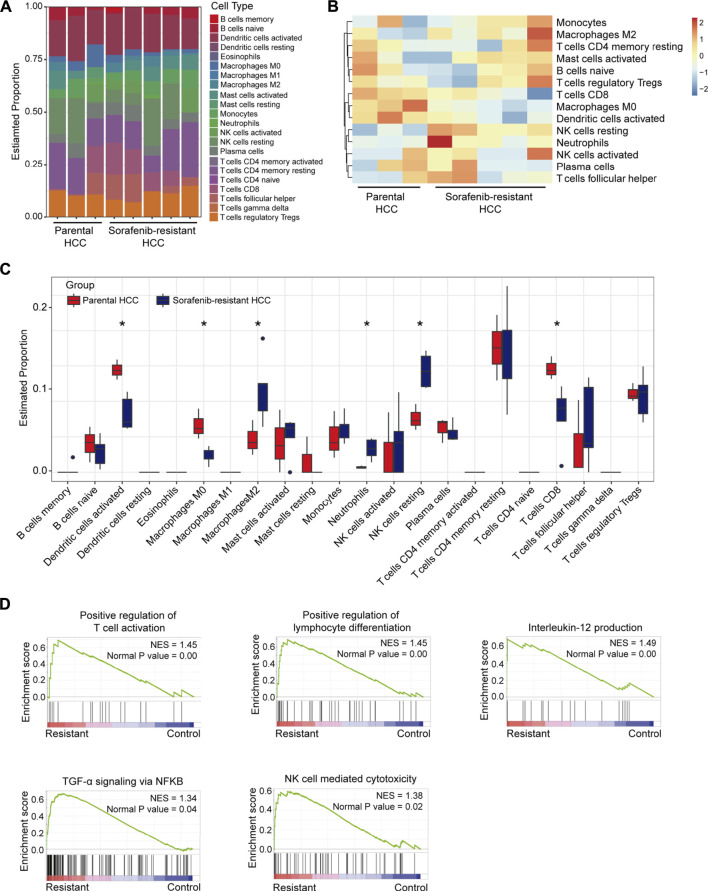
Immunocyte infiltration levels and immune-associated signaling pathways in sorafenib-resistant HCC. **(A)** Infiltration levels of 22 types of immune cells in five sorafenib-resistant and three parental HCC xenografts. **(B)** Infiltration heatmap of major 14 types of immune cells. **(C)** Infiltration differences of 22 types of immune cells between sorafenib-resistant and parental HCC xenografts. **(D)** Enrichment plots showing the immune-associated signaling pathways in sorafenib-resistant HCC cells.

At first, the immune microenvironment in five sorafenib-resistant and three parental HCC xenografts was analyzed. The infiltration levels of 22 immunocyte types between sorafenib-resistant and control HCC cells were explored using the CIBERSORT R package. Immunocyte infiltration percentages of each subtype are presented (Figure 1A), and the heatmap was also constructed to determine infiltration levels of the significant 14 immunocyte types (Figure 1B). The infiltration patterns of immunocytes between sorafenib-resistant and parental HCC xenografts were further explored. The results found that six tumor-infiltrating immunocytes ( CD8^+^ T cells, M0 macrophages, M2 macrophages, neutrophils, resting NK cells, and activated dendritic cells) were associated with sorafenib resistance (Figure 1C). The infiltration of CD8^+^ T cells, M0 macrophages, and activated dendritic cells was downregulated in sorafenib-resistant HCC, while M2 macrophages, neutrophils, and resting NK cells were upregulated. We then performed the GSEA of the expression data based on hallmark gene sets to identify signaling pathways in sorafenib-resistant HCC cells using the GSE94550 dataset. The results revealed that several immune-related signaling pathways, including positive regulation of T cell activation and lymphocyte differentiation, interleukin-12 production, TGF-α signaling *via* NF-κB, and NK cell-mediated cytotoxicity, were enriched (Figure 1D). Meanwhile, sorafenib-mediated pathways are also enriched, including RAB protein signal transduction, apoptosis, and positive regulation of response to endoplasmic reticulum stress (Supplementary Figure S1A).

**FIGURE 2:**
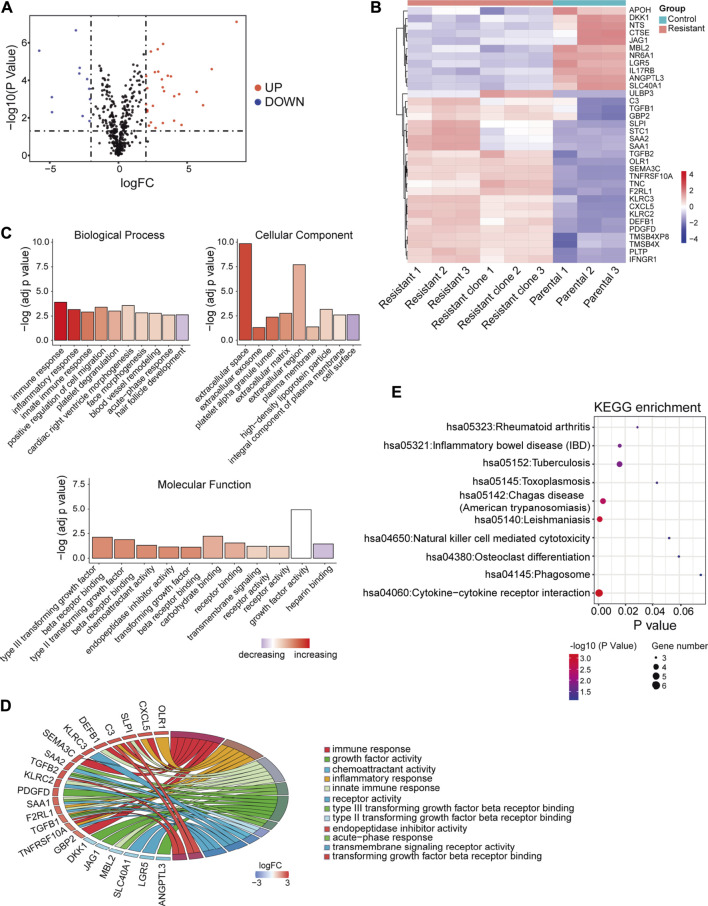
DEIRGs between sorafenib-resistant and control cells and functional enrichment analysis. **(A)** Volcano plot of DEIRGs between sorafenib-resistant and control cells. **(B)** Hierarchical clustering heat maps of DEIRGs. **(C)** Bar plot of enriched GO terms in biological process, cell component, and molecular function. **(D)** GOChord plot indicating the relationship between immune-related GO terms and DEIRGs. The color represents upregulation (red) or downregulation (blue). **(E)** Dot plot showing the enriched KEGG pathways in DEIRGs.

Next, the DEGs between sorafenib-resistant and control cells were also screened. A total of 292 genes were identified as DEGs, comprising 175 upregulated and 117 downregulated genes (Supplementary Figures S1B,C), and overlapped with 2,498 immune-related genes from the ImmPort database. The included 23 upregulated and 11 downregulated genes were further identified as DEIRGs (Table 1). The volcano plots and heat maps were presented (Figures 2A,B), which provides a compendium of gene expression between sorafenib-resistant and control HCC groups. Together, these data indicate dysregulated immune-related profiles in sorafenib-resistant HCC cells.

**TABLE 1:** DEIRGs in sorafenib-resistant HCC cells.

ID	ConMean	ResistMean	LogFC	Adjust *p* value
OLR1	7.458849	16.09118	8.63	7.65E-08
CXCL5	8.301718	15.0951	6.79	2.54E-05
SLPI	5.02033	11.18248	6.16	0.00213
TMSB4XP8	11.10576	16.79371	5.69	0.000411
C3	9.039247	13.62951	4.59	0.0239
TNC	4.868948	9.292524	4.42	0.000547
TMSB4X	11.9336	15.99538	4.06	0.000705
STC1	4.098038	8.150799	4.05	0.0142
DEFB1	5.217232	9.037453	3.82	6.15E-05
PLTP	6.982715	10.57509	3.59	5.70E-05
KLRC3	5.133362	8.454206	3.32	0.000379
SEMA3C	5.409964	8.658344	3.25	1.15E-06
SAA2	5.071765	8.300421	3.23	0.0189
TGFB2	9.72438	12.94728	3.22	0.000235
KLRC2	5.564573	8.726469	3.16	8.88E-05
IFNGR1	8.916841	11.81208	2.9	3.45E-05
PDGFD	4.628085	7.490103	2.86	2.21E-06
SAA1	5.578131	8.279558	2.7	0.0346
F2RL1	6.660483	9.310876	2.65	3.67E-05
TGFB1	8.822688	11.33489	2.51	0.00229
TNFRSF10A	6.300875	8.657604	2.36	2.92E-06
GBP2	6.467234	8.828952	2.36	0.0175
ULBP3	4.888659	7.105909	2.22	0.026
DKK1	12.0003	9.934429	−2.07	0.00101
JAG1	14.50584	12.40063	−2.11	0.0148
MBL2	7.87852	5.745177	−2.13	0.000283
SLC40A1	12.60138	10.32488	−2.28	8.62E-05
APOH	13.59051	10.98391	−2.61	0.00799
LGR5	8.182768	5.3616	−2.82	2.16E-05
IL17RB	8.654049	5.802345	−2.85	4.33E-05
NR6A1	10.7923	7.689057	−3.1	2.17E-07
CTSE	10.63313	5.794716	−4.84	0.00499
NTS	9.470508	4.617572	−4.85	0.00079
ANGPTL3	14.94593	9.171327	−5.77	2.64E-06

### Functional enrichment analyses and mutation signatures of differentially expressed immune-related genes

To explore biological functions of DEIRGs in sorafenib-resistant HCC patients, we conducted functional enrichment analysis using the “GOplot R” package. Based on the GO analysis results, top 10 biological process terms, nine cellular component terms, and 11 molecular function terms are presented (Figure 2C; Supplementary Table S2–S4). Several immune-related GO terms were identified, including “immune response,” “inflammatory response,” “innate immune response,” “type III transforming growth factor beta receptor binding,” “type II transforming growth factor beta receptor binding,” “acute-phase response,” and “transforming growth factor beta receptor binding”. The correlations between 12 immune-related GO terms and corresponding DEIRGs are shown (Figure 2D). The dot plot displayed the 10 KEGG pathways with the enrichment levels of DEIRGs (Figure 2E; Supplementary Table S5).

**FIGURE 3:**
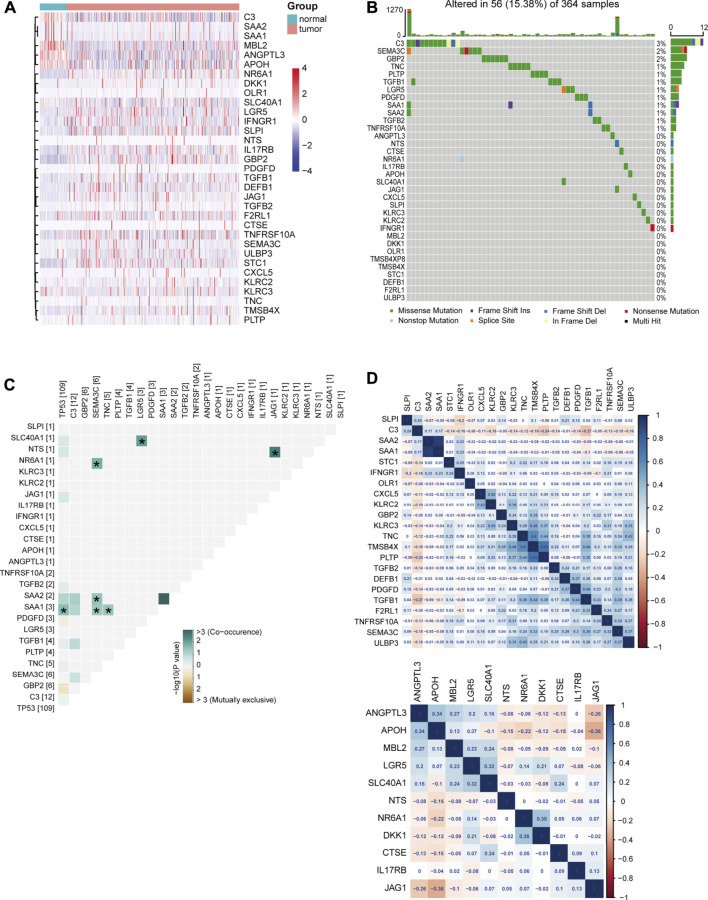
Signature of DEIRGs in TCGA-LIHC. **(A)** Expression heatmap of DEIRGs between normal and LIHC patients in TCGA. **(B)** Mutation status of DEIRGs. **(C)** Correlation between mutation status of DEIRGs and *TP53*. **(D)** Expression correlation between DEIRGs. Upper, upregulated DEIRGs; below, downregulated DEIRGs.

To further explore the underlying signatures of DEIRGs, we constructed a heatmap to show the expression profiles of DEIRGs based on the RNA-seq data of LIHC from TCGA (Figure 3A). The mutation signature of DEIRGs was analyzed using the “maftools R” package. The result showed that 25 DEIRGs displayed mutation and C3 has the highest mutation rate (3%; Figure 3B). *TP53* is the most frequently mutated gene in HCC patients (Yin et al., 2020), and early studies suggested that *TP53* mutation was associated with the response to sorafenib for HCC patients (Gramantieri et al., 2020; Tang et al., 2020). In this study, the correlations between *TP53* and DEIRGs mutations were explored. The results implied that SAA1 is significantly associated with *TP53* mutation (Figure 3C). The correlations of upregulated DEIRGs (upper panel) and downregulated DEIRGs (down panel) were also analyzed (Figure 3D).

### The prognostic features of differentially expressed immune-related genes in TCGA-LIHC samples

**FIGURE 4:**
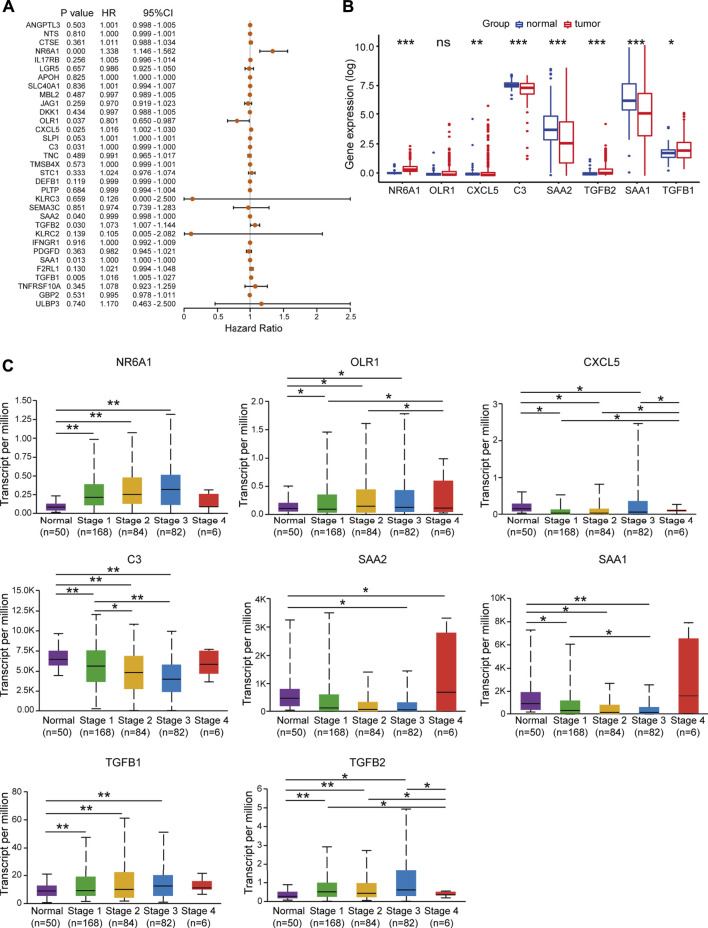
Identification of prognosis risk factors of sorafenib resistance. **(A)** Forest plot showing the prognostic values of DEIRGs in univariate Cox proportional hazards regression analysis. **(B)** Expression levels of eight prognosis risk factors between normal and LIHC patients in TCGA. **(C)** Expression levels of eight prognosis risk factors in LIHC patients in different stages.

Next, univariate Cox regression analysis was performed to identify prognosis-related DEIRGs in LIHC (*p* < 0.05). Eight prognosis risk DEIRGs were identified, including six high-risk DEIRGs (NR6A1, CXCL5, C3, TGFB1, TGFB2, and SAA1) and two low-risk DEIRGs (OLR1 and SAA2; Figure 4A). The expression levels of eight prognosis risk DEIRGs in normal and HCC tissues were analyzed. The results found that NR6A1, CXCL5, TGFB1, and TGFB2 were upregulated, while C3, SAA1, and SAA2 were downregulated. Meanwhile, no significant difference was found for OLR1 (Figure 4B), after which the tissue expression of eight prognosis-related DEIRGs between normal and different TNM-stage HCC was evaluated using the UALCAN database (Figure 4C).

**FIGURE 5:**
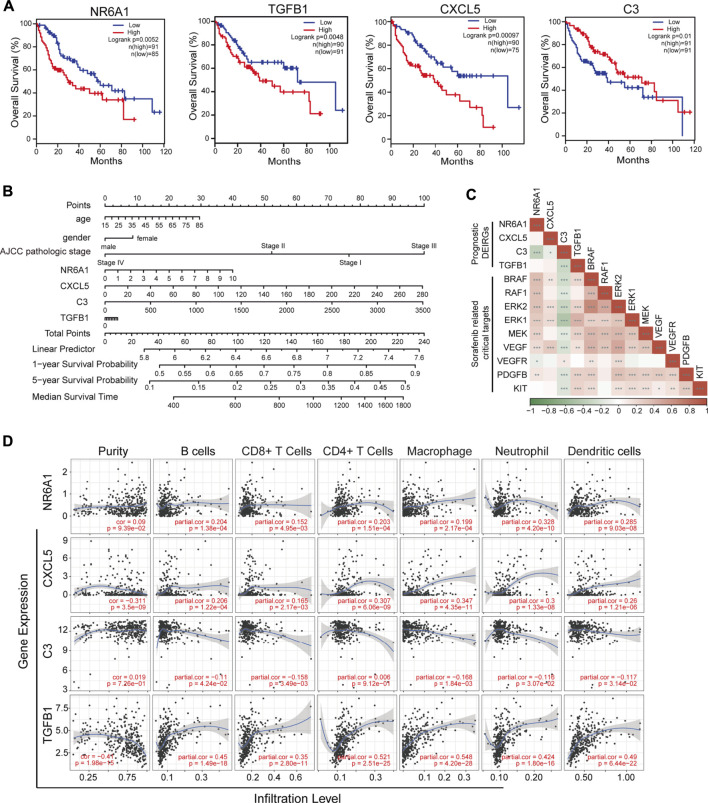
Identification of prognostic-related DEIRGs and immune cell infiltration. **(A)** Kaplan–Meier curve analysis overall survival of NR6A1, TGFB1, CXC5, and C3 in LIHC patients. **(B)** Nomogram model showing four independent prognostic DEIRGs and clinical risk factors in LIHC patients. **(C)** Heatmap of expression correlation between the four prognosis risk factors and nine sorafenib-related downstream targets. **(D)** Correlation between immune cell infiltration and prognostic DEIRGs in LIHC patients.

To validate the prognostic value of eight selected DEIRGs, we performed Kaplan–Meier analysis for overall survival in TCGA-LIHC patients. According to the survival analysis, four prognostic DEIRGs were identified (*p* < 0.05). The results showed highly expressed NR6A1, CXCL5, and TGFB1, along with lower expressed C3 correlated with poor survival (Figure 5A, Supplementary Figure S2A). A nomogram of integrated scores was developed for predicting 1- and 5-year survival and median survival time. Predictors of the nomogram included four independent prognostic DEIRGs ( NR6A1, CXCL5, TGFB1, and C3) and clinical risk factors (including age, gender, and AJCC pathologic stage; Figure 5B). The nomogram model revealed that NR6A1, CXCL5, and C3 were primary risk factors to predict the survival of HCC patients. As a multi-tyrosine kinase inhibitor, sorafenib blocks the RAF-MEK-ERK pathway to inhibit tumor cell proliferation and interacts with vascular endothelial growth factor receptors to attenuate tumor angiogenesis. Therefore, the correlations between four prognostic DEIRGs (including NR6A1, CXCL5, TGFB1, and C3) and sorafenib-mediated nine critical downstream targets ( BRAF, RAF1, ERK1, ERK2, MEK, VEGF, VEGFR, PDGFB, and KIT) were individually analyzed in TCGA-LIHC patients. The results revealed that the expression of NR6A1, CXCL5, and TGFB1 was positively correlated with the sorafenib-related key targets, while C3 was negatively related with the sorafenib-related key targets (Figure 5C).

### Immune infiltration and single-cell expression of four prognosis-related differentially expressed immune-related genes

The tumor immune estimation resource (TIMER) platform was applied to explore the correlation between infiltration levels of immune cells and the expression of four prognosis-related genes (including NR6A1, CXCL5, TGFB1, and C3). The results demonstrated that CXCL5 and TGFB1 were significantly associated with purity (*p* < 0.05, correlation = −0.311 and −0.41, respectively). Increased expression of NR6A1, CXCL5, and TGFB1 was positively correlated with elevated immune infiltration level (*p* < 0.05); while high C3 expression was negatively associated with the infiltration of B cells, CD8^+^ T cells, macrophages, neotrophils, and dendritic cells (*p* < 0.05; Figure 5D). Different cell type expression of four selected DEIRGs in the liver was investigated using The Single Cell Type Atlas. The UMAP plots and bar charts showed that NR6A1 and CXCL5 were mainly expressed in cholangiocytes, C3 in hepatocytes, and TGFB1 in T cells, Kupffer cells, Ito cells, and endothelial cells (Supplementary Figures S2B,C), indicating the potential roles of various immunocytes in sorafenib resistance.

### Immune cell infiltration analysis in the sorafenib-resistant mouse model

**FIGURE 6:**
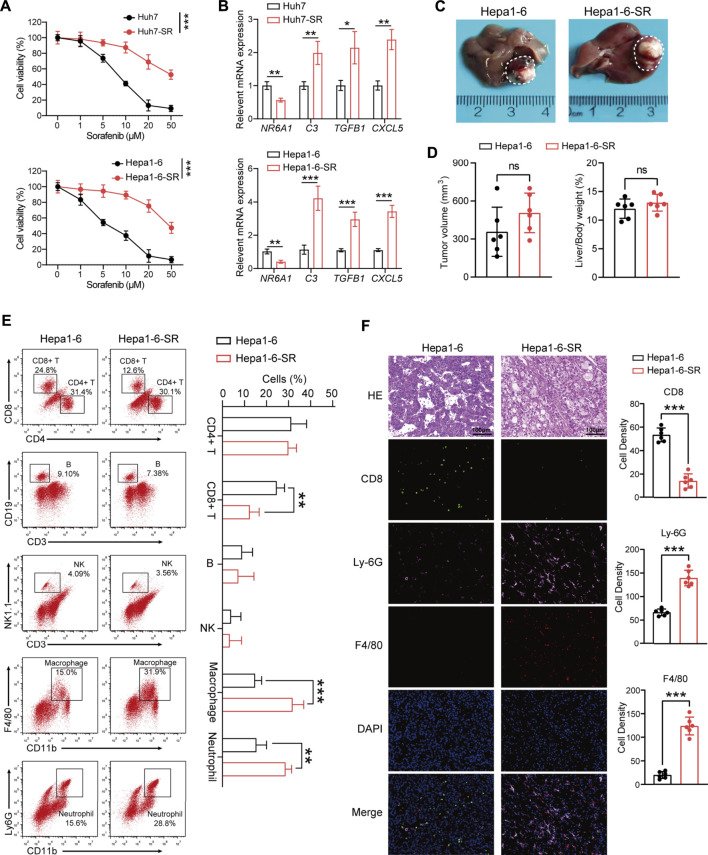
Tumor immune microenvironment analysis in the sorafenib-resistant HCC mouse model. **(A)** Two sorafenib-resistant HCC cell lines (Huh7-SR and Hepa1-6-SR) were established and confirmed by CCK-8 assay. **(B)** Expression values of four prognostic DEIRGs in sorafenib-resistant HCC cells. **(C)** Representative images of the liver from a orthotopic xenograft sorafenib-resistant and control HCC mouse model. **(D)** Tumor volume comparison between the control and sorafenib-resistant HCC mouse model. **(E)** Representative immunocyte infiltration analysis using FCM in the mouse model. **(F)** Multiplex immunofluorescence staining CD8, Ly-6G, and F4/80 in control and sorafenib-resistant HCC tissues.

To validate database results, we established a sorafenib-resistant human HCC cell line (Huh7-SR) and mouse HCC cell line (Hepa1-6-SR). The drug resistance of the two cell lines was confirmed using the CCK-8 assay (Figure 6A). NR6A1 was downregulated in sorafenib-resistant cells, while C3, TGFB1, and CXCL5 were upregulated (Figure 6B), which were consistent with former sequencing analysis (Regan-Fendt et al., 2020). To investigate the TIME *in vivo*, we constructed live orthotopic xenograft mice using Hepa1-6 or Hepa1-6-SR cells (Figure 6C). No significant differences were observed for tumor volume and liver/body weight ratio between the two groups (Figure 6D). The immune cell infiltration in tumor tissues was analyzed by FCM (Supplementary Figures S3A,B). The results identified low infiltration levels of CD8^+^ T cells and high levels of macrophages and neutrophils in sorafenib-resistant mouse HCC tissues. However, no significant infiltration differences were exhibited for CD4^+^ T cells, B cells, and natural killer (NK) cells (Figure 6E). The mIF of HCC tissues was further performed. The results showed that CD8 was attenuated in sorafenib-resistant tissues, but Ly-6G (neutrophils marker) and F4/80 (macrophages marker) were highly expressed (Figure 6F). These results help us obtain a clear understanding concerning the TIME changes in sorafenib-resistant HCC tissues.

### Nalidixic acid is an antagonist for sorafenib-resistant hepatocellular carcinoma

**FIGURE 7:**
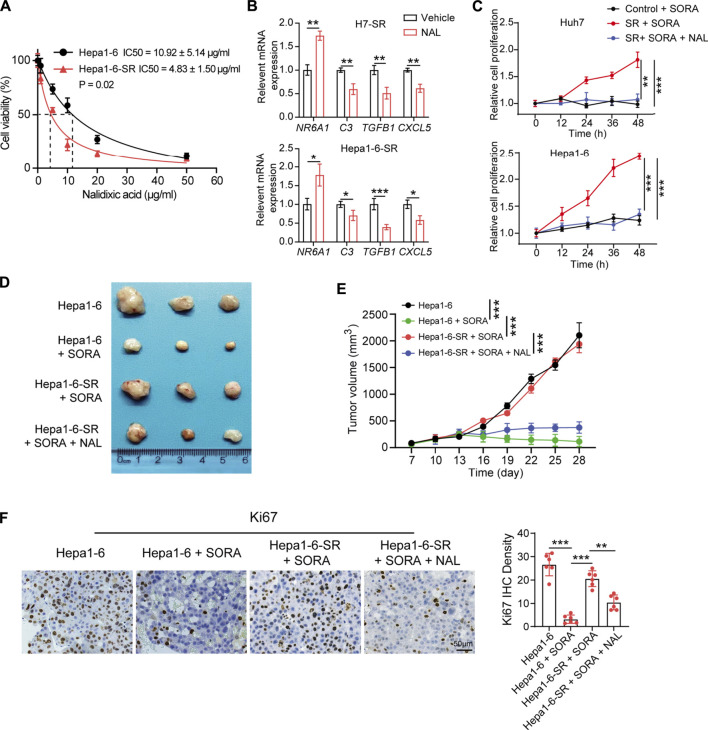
Nalidixic acid overcomes sorafenib resistance and inhibits HCC development. **(A)** Cell viability showing the IC_50_ concentrations of nalidixic acid treatment in Hepa1-6-SR and parental Hepa1-6 cells. **(B)** Expression changes of four prognostic DEIRGs ( NR6A1, CXCL5, C3, and TGFB1) in sorafenib-resistant HCC cells treated with nalidixic acid. **(C)** CCK-8 assay assessed cell viability in Huh7-SR and Hepa1-6-SR cells treated with sorafenib or sorafenib plus nalidixic acid. **(D)** Representative photos of tumors presented after 4 weeks of different treatments. **(E)** Tumor growth curves of different treatments in the sorafenib-resistant HCC mouse model. **(F)** IHC staining of Ki67 in different treatment groups.

The CMap provides a convenient strategy for revealing the connections among small molecules, genetic signatures, and diseases (Lamb et al., 2006). Next, the CMap was utilized to screen potential small molecules for sorafenib-resistant HCC according to the expression of DEIRGs. Fifteen small-molecule drugs were identified according to the screening criteria (absolute mean value > 0.4 and *p* < 0.01). Nalidixic acid (NAL) identified as a prominent drug, which negatively correlated with the expression of DEIRGs in sorafenib-resistant HCC cells (Table 2). Therefore, the treatment effect of NAL was evaluated in sorafenib-resistant HCC cells. The results showed that Hepa1-6-SR cells had lower IC_50_ than Hepa1-6 cells (Figure 7A), indicating more sensitivity for NAL treatment. When treated with NAL, the expression of NR6A1 was upregulated, while those of CXCL5, C3, and TGFB1 were downregulated in Huh7-SR and Hepa1-6-SR cells (Figure 7B). Next, the cell proliferation rate was explored using the CCK-8 assay. The results suggested that the combination of NAL and sorafenib effectively inhibited proliferation of Huh7-SR and Hepa1-6-SR cells (Figure 7C). The treatment effect of NAL was further explored using subcutaneous xenograft mice *in vivo*. The group treated with NAL and sorafenib had smaller volume and lower growth rate than other groups (Figures 7D,E). Moreover, the Ki67 staining exhibited that tissue sections from the synergical NAL and sorafenib group showed a lower proportion of proliferation cells than in other groups (Figure 7F). These evidences suggest that NAL can reverse sorafenib resistance and inhibit sorafenib-resistant HCC cell progression *in vitro* and *in vivo*.

**TABLE 2:** Results of CMap analysis.

Small molecule	Mean score	n^a^	Enrichment	*p* value	Specificity	Percent non-null^b^
Nalidixic acid	−0.813	5	−0.934	0	0	100
MG-262	−0.676	3	−0.917	0.00096	0.0709	100
Lasalocid	0.598	4	0.839	0.00103	0.0245	100
Butyl hydroxybenzoate	−0.428	5	−0.774	0.00106	0.0068	80
Etynodiol	0.574	4	0.834	0.00115	0	100
Aceclofenac	−0.5	4	−0.835	0.00131	0	100
Colforsin	0.447	5	0.766	0.0016	0.0101	60
Hydrastinine	−0.402	5	−0.74	0.00234	0.0049	60
Sisomicin	0.415	4	0.812	0.00237	0	75
Chlortetracycline	−0.447	5	−0.74	0.00242	0	80
Digoxigenin	0.455	5	0.741	0.00268	0.0614	80
Benzthiazide	−0.435	4	−0.8	0.00316	0.0102	75
11-deoxy-16, 16-dimethyl prostaglandin E2	0.462	4	0.774	0.00495	0.0245	75
Praziquantel	−0.456	4	−0.767	0.00585	0	75
Piracetam	−0.475	4	−0.744	0.00851	0.0122	75

a n: The matching result number of each small molecule applied in different concentration and cell lines.

b Percent non-null: The percentage of matching score which is not 0.

## Discussion

Sorafenib can block the proliferation and angiogenesis of tumor cells and has been recommended as the first-line regimen for advanced unresectable HCC patients (Heimbach et al., 2018). Researchers also found that the antitumor effects of sorafenib in other tumors, such as prostate cancer, myeloid leukemia, renal cell carcinoma, and desmoid tumor (Escudier et al., 2007; Kharaziha et al., 2012; Gounder et al., 2018; Burchert et al., 2020). Unfortunately, clinical trials indicated that overall survival time was slightly prolonged for HCC patients treated with sorafenib compared with patients receiving placebo (Llovet et al., 2008; Cheng et al., 2009). The main reason for the decrease is HCC heterogeneity and sorafenib resistance (Zhu et al., 2017). Thus, it is urgent to explore resistant mechanisms and evaluate novel synergistic drugs for sorafenib-resistant HCC patients.

Previous studies showed that overexpressed EGFR and its downstream targets, especially Ras, Raf, MEK, and ERK, might predict inadequate sorafenib response (Ezzoukhry et al., 2012). Additionally, a high circulating level of miR-30e-3p suggested the development of sorafenib resistance (Gramantieri et al., 2020). By CRISPR/Cas9 library screening, researchers found that activation of phosphoglycerate dehydrogenase was positively correlated with sorafenib resistance (Wei et al., 2019). Furthermore, CD24 was reported to be upregulated in sorafenib-resistant HCC cell lines, and its depletion led to a significant increase for sorafenib efficacy (Lu et al., 2018). Also, researchers found that part of adverse events occurred during the application of sorafenib, which indicated a better prognosis for HCC patients (Granito et al., 2016). In addition, the tumor microenvironment also plays a critical role in sorafenib response. Hypoxia in solid tumors is often related with chemotherapy failure, including sorafenib (Méndez-Blanco et al., 2018). Previous studies have also shown that overexpressed hypoxia inducible factor-1α in hypoxic cells regulated various hypoxia-related gene expression to induce sorafenib resistance (Liu et al., 2012). Moreover, Zhou *et al.* proposed that highly expressed CCL2 and CCL17 in tumor-associated neutrophils promoted the infiltration of macrophages and regulatory T cells, thus compromising sorafenib efficacy in HCC (Zhou et al., 2016).

In this study, we assessed immune cell infiltration levels between sorafenib-resistant and parental HCC xenograft using CIBERSORT. The results showed that infiltration levels of M2 macrophages, neutrophils, and resting NK cells were increased, while CD8^+^ T cells, M0 macrophages, and activated dendritic cells were less infiltrated in sorafenib-resistant HCC. We further confirmed that high levels of macrophages and neutrophils and low levels of CD8^+^ T cells in the sorafenib-resistant mouse HCC model were observed. Moreover, GSEA results showed that several immune pathways are highly enriched in sorafenib-resistant cells. These results proved that sorafenib resistance was highly associated with dysfunction of immune cells and signaling pathways.

According to recent studies, immune checkpoints and cytokines are closely related to sorafenib resistance (Liu and Qin, 2019). Chen *et al.* found that combinational anti−PD-1 antibody and sorafenib provide a promising option for small subsets of HCC patients (Chen et al., 2016). Furthermore, upregulated CCL22 could forcefully promote sorafenib resistance in HBV-associated HCC (Gao et al., 2020). Here, we analyzed the expression profiles between sorafenib-resistant and control HCC cells and identified 33 DEIRGs. TCGA-LIHC database analysis showed that the selected 33 DEIRGs have a lower mutation rate in HCC patients, in which only SAA1 mutation significantly co-occurred with *TP53* mutation. Further univariate Cox regression and Kaplan–Meier analysis identified that the expression levels of NR6A1, CXCL5, C3, and TGFB1 were significantly associated with overall survival time of HCC patients. TIMER database results showed NR6A1, CXCL5, C3, and TGFB1 were positively associated with immunocyte infiltration.

NR6A1, a nuclear hormone receptor family member, regulated lipogenesis through mTORC1 in HepG2 cells (Wang et al., 2019). However, the relationship between NR6A1 and immunocyte infiltration was less known. CXCL5 is one of the critical proinflammatory chemokines in the TME (Zhang et al., 2020). It can mediate immune cell infiltration and promote angiogenesis, tumor growth, and metastasis by binding to its receptor, C-X-C motif chemokine receptor 2 (CXCR2) (Zhou et al., 2012; Romero-Moreno et al., 2019). In HCC, Zhou et al. found that stem-like cells secreted high levels of CXCL5 to recruit neutrophil infiltration, and increased CXCL5 expression is associated with poor survival (Zhou et al., 2019). It has been reported that TGFB1 was involved in regulating autophagy in tumors (Nüchel et al., 2018; Liang et al., 2020). In addition, researchers found that sorafenib can alleviate hepatic fibrogenesis by inhibiting TGFB1 expression in the 3D co-culture model of fatty hepatocyte and hepatic stellate cells (Romualdo et al., 2021). C3 is an essential component of innate immune system and participates in detecting and clearing potential pathogens in hosts (Delanghe et al., 2014). Highly expressed C3 was found in tumor metastatic models (Boire et al., 2017) and associated with tumor growth (Aykut et al., 2019).

Few systemic therapies have been shown to improve survival time in advanced HCC patients who fail to respond to sorafenib. A clinical trial showed that regorafenib can significantly improve overall survival time than placebo for HCC patients with limited therapy response to sorafenib (Bruix et al., 2017). Regorafenib can act on multiple targets involved in angiogenesis, cell proliferation, and modulate antitumor immunity in HCC (Granito et al., 2021). In this study, we explored the potential molecular drugs that might be effective for sorafenib-resistant HCC patients using the CMap. Among the identified 15 drugs, a highly negative correlation between NAL and the expression of DEIRGs was found. The *in vitro* and *in vivo* studies showed NAL effectively inhibited sorafenib-resistant HCC cells. These findings suggested that NAL was a promising antagonist for sorafenib-resistant HCC treatment. It should be noted that there are still limitations. First, our research data were originally explored by bioinformatics analysis using public resources, and large sample sizes and further experiments are needed to validate our findings. Second, the DEIRGs of sorafenib resistance are valuable to examine the significance of key risk factors, thus predicting therapy response in real-world HCC patients receiving sorafenib. Third, NAL was commonly used as an antibiotic and anti-inflammatory agent (Luo et al., 2022). However, the exact antitumor mechanism and immune microenvironment changes mediated by NAL in sorafenib-resistant HCC patients need further investigation.

In summary, our study explored the TIME in sorafenib-resistant HCC and found low levels of CD8^+^ T cell infiltration along with high levels of macrophages and neutrophils. NR6A1, CXCL5, C3, and TGFB1 were critical DEIRGs in sorafenib-resistant cells, which were markedly associated with the survival time of HCC patients and infiltration levels of immune cells. Finally, the therapeutic effect of NAL was explored, which might serve as an adjuvant drug for sorafenib-resistant HCC treatment. These results may help researchers learn the detailed mechanism of drug resistance and facilitate identifying therapeutic targets for HCC patients.

## Data Availability

The datasets presented in this study can be found in online repositories. The names of the repository/repositories and accession number(s) can be found in the article/Supplementary Material.
