# Supplementary figures and images for: Nalidixic acid potentiates the antitumor activity in sorafenib-resistant hepatocellular carcinoma via the tumor immune microenvironment analysis
Source: Front Pharmacol. 2022 Aug 22;13:952482. doi: 10.3389/fphar.2022.952482 (PMC9441713; doi:10.3389/fphar.2022.952482)

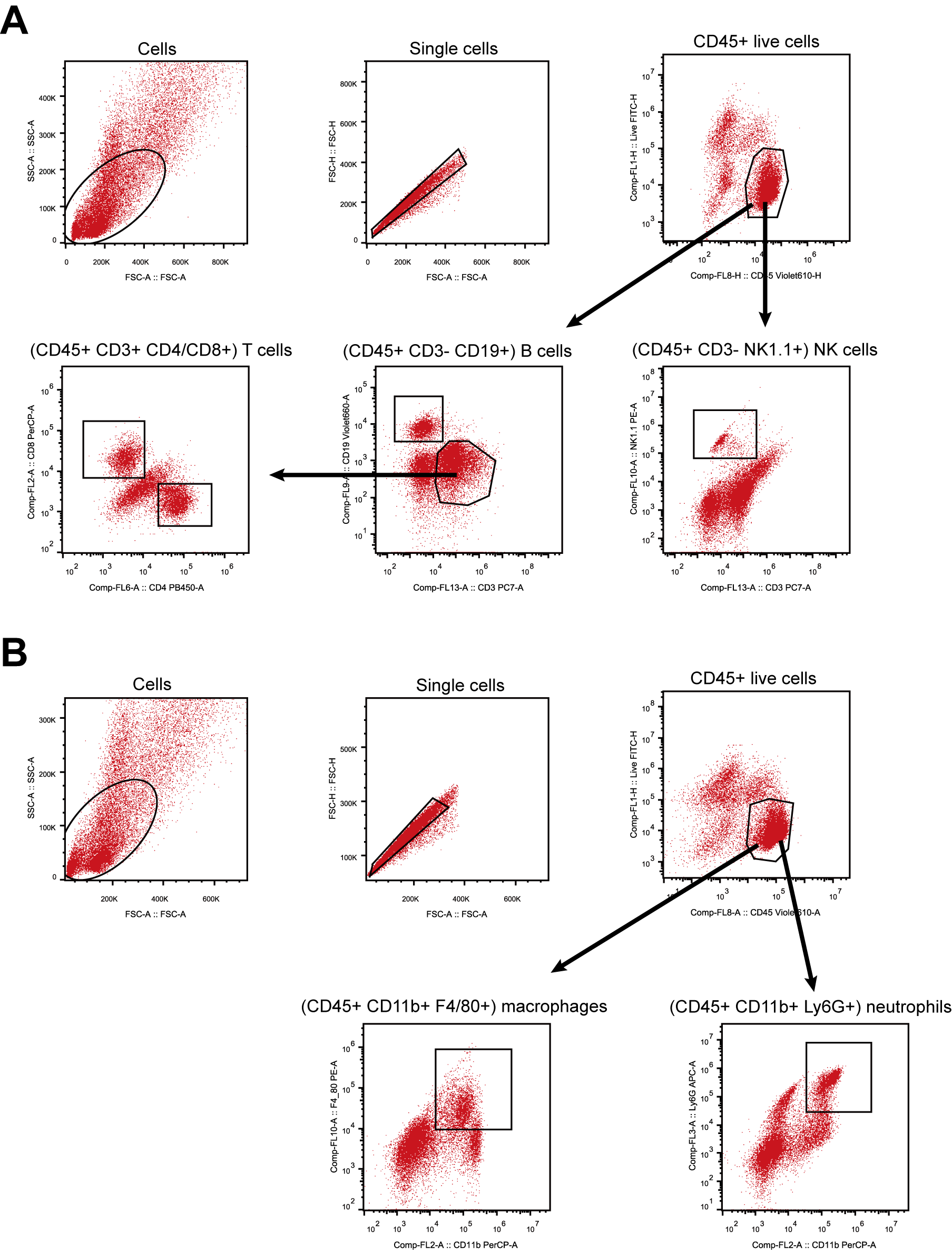

Supplement: Supplementary file 3 [file Image3.TIF]

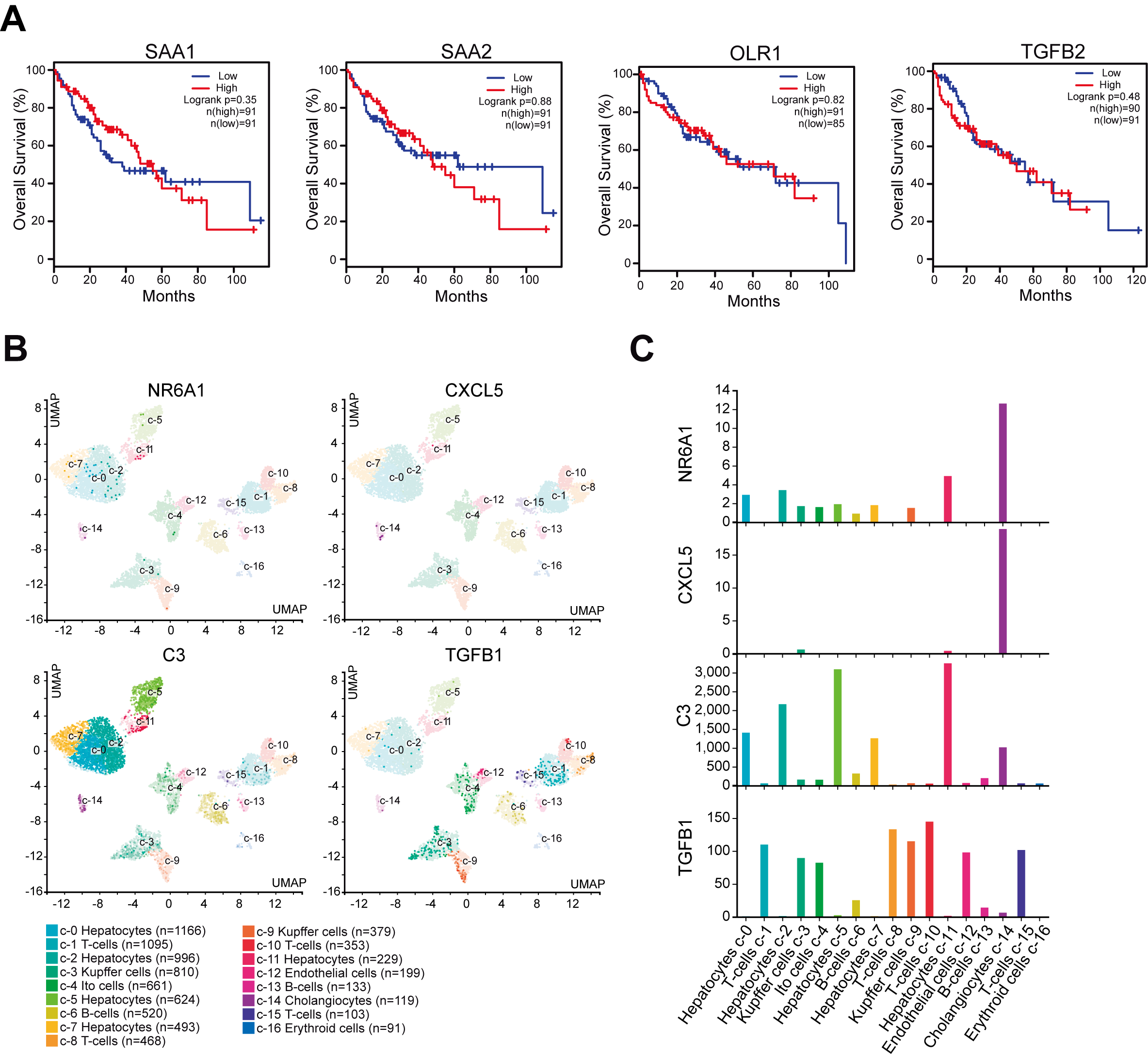

Supplement: Supplementary file 4 [file Image2.TIF]

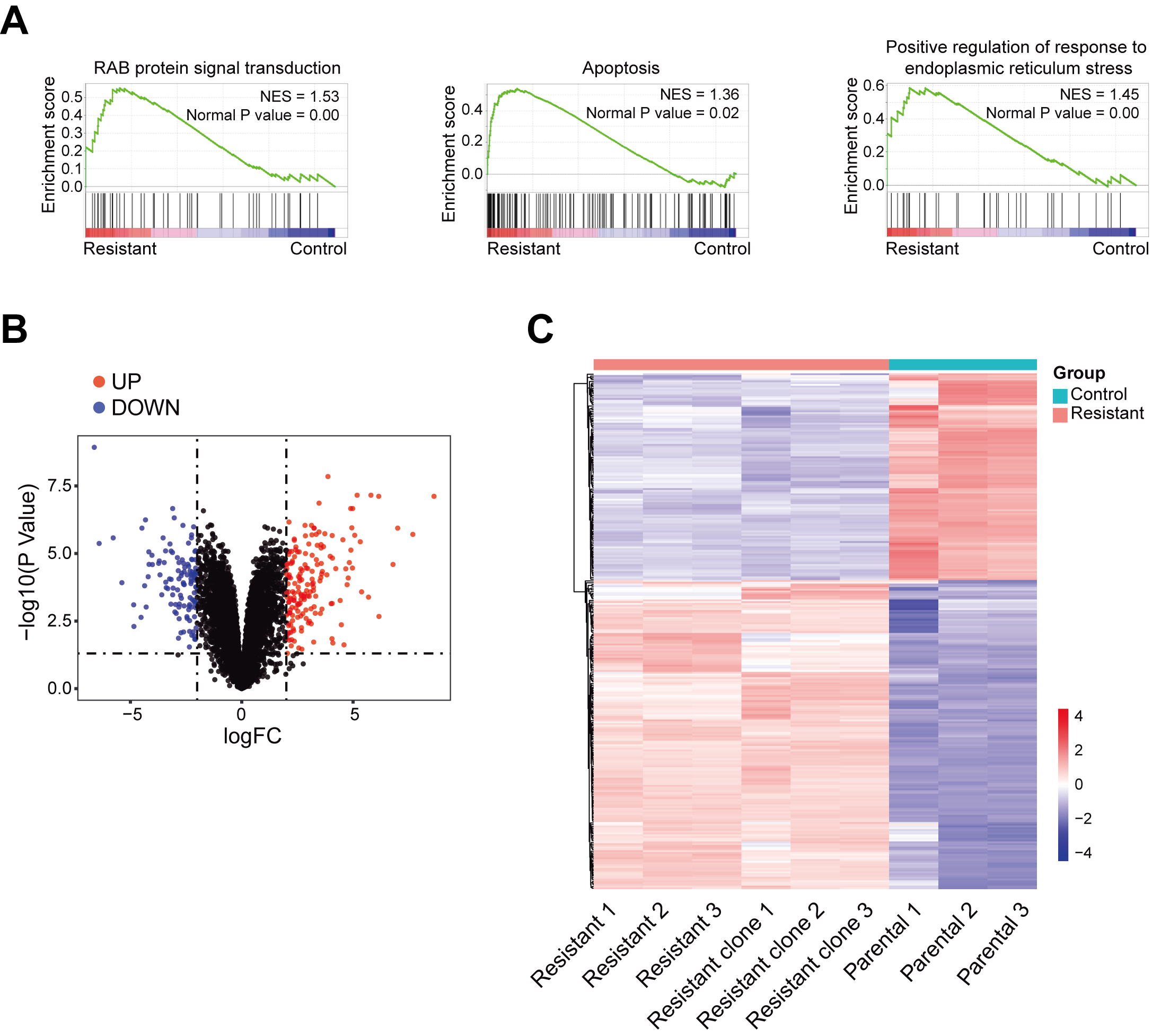

Supplement: Supplementary file 5 [file Image1.TIF]
