# Supplementary material for: Nalidixic acid potentiates the antitumor activity in sorafenib-resistant hepatocellular carcinoma via the tumor immune microenvironment analysis
Source: Front Pharmacol. 2022 Aug 22;13:952482. doi: 10.3389/fphar.2022.952482 (PMC9441713; doi:10.3389/fphar.2022.952482)
Supplement: Supplementary file 8 [file Table6.DOCX]

**FIGURE S1** GSEA analysis and DEGs identification of sorafenib resistance. **(A)** sorafenib mediated signal pathways enriched by GSEA analysis. **(B)** The volcano plot of DEGs. **(C)** The hierarchical clustering heat maps of DEGs.

**FIGURE S2** Survival and single-cell expression analysis of indicated DEIRGs. **(A)** Kaplan-Meier curve analyses overall survival of SAA1, SAA2, OLR1, and TGFB2 in LIHC patients. **(B)** The single-cell expression of 4 prognostic DEIRGs in the liver. **(C)** The histograms of 4 prognostic DEIRGs expression levels in multiple cell types.

**FIGURE S3** FCW analysis flow for immune cell infiltration. **(A)** FCW panel A analysis flow for CD4^+^ T cells, CD8^+^ T cells, B cells, and NK cells. **(B)** FCW panel B analysis flow for macrophages and neutrophils.
